# Oral health-related impact profile of patients treated with fixed, removable, and telescopic dental prostheses in student courses—a prospective bicenter clinical trial

**DOI:** 10.1007/s00784-020-03532-w

**Published:** 2020-08-27

**Authors:** Anja Liebermann, K. Erdelt, I. Lente, D. Edelhoff, M. Schmitter, A. Winter

**Affiliations:** 1 Department of Prosthetic Dentistry, LMU Munich, Goethestrasse 70, 80336 Munich, Germany; 2 Department of Prosthetic Dentistry, Würzburg, Germany

**Keywords:** OHIP-49, OHIP-53, Student course, Removable dental prosthesis, Fixed dental prosthesis, Telescopic dental prosthesis

## Abstract

**Objectives:**

To analyze the oral health-related impact profile in patients treated with three different types of dental prosthesis in student courses.

**Materials and Methods:**

This prospective bicenter clinical trial was conducted with 151 patients being treated with fixed (*n* = 70), removable (*n* = 61), or telescopic dental prostheses (*n* = 20) in clinical student courses of two German universities from October 2018 to October 2019. All patients completed three standardized German versions of the Oral Health Impact Profile (OHIP-G49/53) before prosthetic treatment (T0), at control after 1 week (T1), and after 3 months (T2), divided into five dimensions: (a) appearance, (b) oral function, (c) psychosocial impact, (d) linguistic limitations, and (e) orofacial pain. Data were analyzed with Kolmogorov–Smirnov, Wilcoxon signed-rank, Kruskal–Wallis, Mann–Whitney, and Cronbach’s alpha tests.

**Results:**

Within T0–T1 and T0–T2, greater improvements were determined for removable compared with fixed dental prostheses for the dimensions’ oral function (*p* ≤ 0.014), linguistic limitations (*p* ≤ 0.016), and appearance (*p* ≤ 0.003). No significant differences were found between fixed and telescopic dental prostheses (*p* ≥ 0.104) or between removable (partial dental prosthesis with clasps and complete dental prosthesis) and telescopic dental prostheses (*p* ≥ 0.100). Within T1–T2, a significant improvement in orofacial pain could be determined (*p* = 0.007).

**Conclusions:**

Restorations presented an improvement in oral health-related quality of life. Removable dental prostheses showed better improvement than fixed ones in various dimensions.

**Clinical relevance:**

Knowledge about the influence of oral health-related quality of life on the three different types of prosthesis used in student courses can be of decisive help in dental consultations.

## Introduction

Overall quality of life (QoL) depends crucially on the oral health-related quality of life (OHRQoL) [1]. The individual analysis of OHRQoL and clarification of the influence of a prosthetic restoration on patients’ OHRQoL can usually be analyzed using an Oral Health Impact Profile (OHIP) questionnaire [2, 3]. OHIP questionnaires with varying numbers of subjective questions—OHIP-12 to OHIP-53—are available in the different national languages [2–14]. The oral health-related impact profile is considered multidimensional, and all questions are summarized by different dimensions to identify the specific influences and thus reference a higher-order factor [10]. These are usually subdivided into four highly correlated factors—oral function, orofacial pain, orofacial appearance, and psychological impact [2, 3, 10], but may also undergo modification [14, 15].

In general, an OHIP questionnaire represents a subjective evaluation of individual oral health and, in some cases, of patients’ expectations, feelings, and satisfaction with regard to a prosthetic restoration [2, 3, 10, 16]. Consequently, depending on the study design, there may be great variations between different studies.

The scientific literature generally shows an improvement in oral health-related impact profile through prosthetic restorations, although this appears to depend on the restoration type [11]. In two studies on prosthetic restorations, a significant improvement in oral health-related impact profile was recorded for patients with complete dental prostheses; one of the studies also investigated patients in student courses [4, 17]. In addition, treatment with different double crown removable dental prostheses improves the oral health-related impact profile [18]. Patients with removable dental prostheses, however, show a worse oral health-related impact profile than patients with fixed restorations [12].

To the best of the authors’ knowledge, there has been no comparable study of the oral health-related impact profile of patients who have been prosthetically restored with three different types of dental prosthesis in student courses and comparison between them. The hypotheses of the present study state that:

1. There is no change in patients’ oral health-related impact profile after prosthetic restoration in a student course in relation to specific questions and the dimensions.
2. There are no differences in patients’ oral health-related impact profile between prosthetic type after prosthetic treatment within a student course.

## Materials and methods

**Fig. 1:**
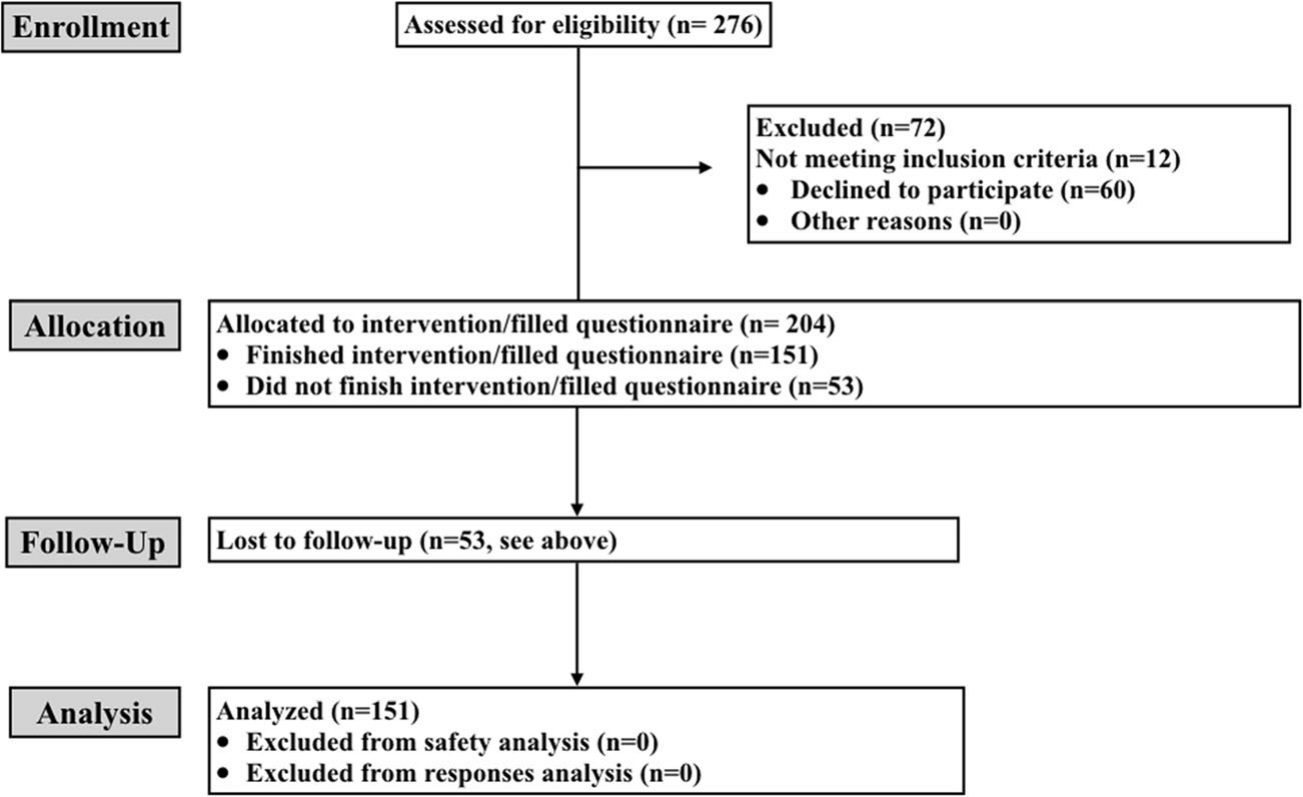
Consort flow diagram for clinical study

The present prospective bicenter clinical trial was conducted in two prosthetic dentistry departments in Germany. The ethics committees of both universities approved the study (approval numbers 18-482 and 139/18). In advance, written informed consent was obtained from all patients participating. A Consort flow diagram is presented in Fig. 1, where eligible, excluded, and included patients are listed.

A total of 151 patients were included (Munich *n* = 75; Würzburg *n* = 76); they were treated with fixed (*n* = 70), removable (*n* = 61), or telescopic (*n* = 20) dental prostheses (Table 1).

**Table 1:** Overview of descriptive patient data including age, gender, and restoration types

	Total number	Total number Center 1	Total number Center 2
Age (years) T0	Total: 64.7 ± 10.5 Male: 64.9 ± 10.5 Female: 64.5 ± 10.6 Without restorations: 64.6 ± 10.4 Fixed restorations: 64.5 ± 11.6 Removable dental prosthesis: 64.4 ± 10.6 Telescopic dental prosthesis: 64.3 ± 9.80	Total: 65.5 ± 11.3 Male: 65.5 ± 11.3 Female: 65.4 ± 11.8 Without restorations: 65.5 ± 11.3 Fixed restorations: 65.1 ± 11.5 Removable dental prosthesis: 67.3 ± 10.9 Telescopic dental prosthesis: 65.9 ± 10.3	Total: 63.5 ± 9.68 Male: 63.9 ± 9.70 Female: 63.8 ± 9.82 Without restorations: 63.4 ± 9.30 Fixed restorations: 64.4 ± 10.0 Removable dental prosthesis: 63.8 ± 9.80 Telescopic dental prosthesis: 63.6 ± 9.70
Gender (number)	Male: 80 Female: 71	Male: 39 Female: 36	Male: 41 Female: 35
Restoration type T0 (number)	Without restorations: 52 Fixed restorations: 55 Removable dental prosthesis: 43 Telescopic dental prosthesis: 1	Without restorations: 21 Fixed restorations: 36 Removable dental prosthesis: 19 Telescopic dental prosthesis: 1	Without restorations: 31 Fixed restorations: 19 Removable dental prosthesis: 24 Telescopic dental prosthesis: 0
Restoration type T1 (number)	Fixed restorations: 70 Removable dental prosthesis: 61 Telescopic dental prosthesis: 20	Fixed restorations: 38 Removable dental prosthesis: 34 Telescopic dental prosthesis: 5	Fixed restorations: 32 Removable dental prosthesis: 27 Telescopic dental prosthesis: 15
Change of restoration type (number)	0 to 1: 26 0 to 2 : 23 0 to 3: 3 1 to 1: 40 1 to 2: 12 1 to 3: 3 2 to 1: 4 2 to 2: 25 2 to 3: 14 3 to 1: 0 3 to 2: 1 3 to 3: 0	0 to 1: 7 0 to 2 : 12 0 to 3: 2 1 to 1: 28 1 to 2: 8 1 to 3: 0 2 to 1: 3 2 to 2: 13 2 to 3: 3 3 to 1: 0 3 to 2: 1 3 to 3: 0	0 to 1: 19 0 to 2 : 11 0 to 3: 1 1 to 1: 12 1 to 2: 4 1 to 3: 3 2 to 1: 1 2 to 2: 12 2 to 3: 11 3 to 1: 0 3 to 2: 0 3 to 3: 0

*Center 1*, Munich; *Center 2* Würzburg

Without restoration 0; fixed restoration 1; removable dental prosthesis 2; telescopic dental prosthesis 3

All patients were treated in the clinical student courses (students in the 4th and 5th study year) in both centers from October 2018 to October 2019. Fixed dental prostheses included crowns and fixed dental prostheses, removable dental prostheses included partial dental prostheses with clasps and complete dental prostheses, and telescopic dental prostheses were fabricated with friction telescopes. The following inclusion criteria were defined:

1. The patient is at least 18 years old
2. The patient is suitable for treatment in the student course
3. The patient should have good oral hygiene
4. No relocation of the patient is planned within the next 6 months
5. The patient has no known allergies to dental materials

### OHIP-G49/G53 questionnaire

All patients completed a total of three standardized German versions of the Oral Health Impact Profile (OHIP-G49/53). Patients who were treated with removable dental prostheses completed the entire questionnaire with all 53 questions. Patients with fixed dental prostheses completed only 49 questions, as the last three questions are only for removable dental prostheses. Only patients who completed all three questionnaires were included in the study.

The first questionnaire (T0) was completed as a baseline questionnaire before the start of the prosthetic treatment. The second questionnaire (T1) was completed after definitive insertion of the dental prosthesis, at the follow-up appointment approximately 1 week after insertion, and the third questionnaire (T3) was completed after 3 months of use. In the OHIP-G49/53 questionnaire used, patients had five options to answer the questions, and answers were therefore numbered 1 (never) to 5 (very often). All data were recorded in an Excel sheet.

The OHIP-G49/53 questionnaires used were divided by the authors into five dimensions according to Liebermann et al. [15]: (a) appearance, (b) oral function, (c) psychosocial impact, (d) linguistic limitations, and (e) orofacial pain. All questions of the standardized questionnaires used with the specific allocations to the different dimensions are listed in Table 2.

**Table 2:** All five dimensions with related questions of the German OHIP-49/53 (one doubled question), including grouped median (statistical difference) and IQR for all three prosthetic restoration types

Dimension	OHIP-G49/53 question no.	Specific question (translated German questions)	Fixed dental prosthesis			Removable dental prosthesis			Telescopic dental prosthesis		
			Median/IQR T0–T1	Median/IQR T0–T2	Median/IQR T1–T2	Median/IQR T0–T1	Median/IQR T0–T2	Median/IQR T1–T2	Median/IQR T0–T1	Median/IQR T0–T2	Median/IQR T1–T2
Appearance	3 + 10 (doubled question)	Have you felt that your appearance has been affected because of problems with your teeth, mouth, or dental prosthesis?	0.393/1.00	0.316/1.00	− 0.462/0.000	0.971/1.50	0.488/1.00	− 0.018/0.000	0.714/1.00	0.733/1.00	0.111/0.000
	18	Have you avoided smiling because of problems with your teeth, mouth, or dental prosthesis?	0.293/1.00	0.258/1.00	− 0.048/0.000	0.750/2.00	0.750/2.00	0.000/0.000	0.308/1.75	0.333/1.00	− 0.118/0.000
	47	Have you noticed a tooth that does not look right?	0.186/0.000	0.161/0.000	− 0.017/0.000	0.298/1.00	0.310/1.00	0.080/0.000	0.375/1.00	0.692/1.00	− 0.111/0.000
	49	Have you felt uncomfortable about the appearance of your teeth, mouth, or dental prosthesis?	0.164/0.000	0.155/0.000	0.000/0.000	0.438/1.00	0.533/1.00	0.089/0.000	0.125/0.000	0.188/0.000	0.059/0.000
Oral function	1	Have you had difficulty chewing any foods because of problems with your teeth, mouth, or dental prosthesis?	0.500/1.00	0.597/1.00	0.113/0.000	0.971/2.00	1.304/3.00	0.226/1.00	1.44/2.75*	1.82/2.00*	0.571/1.75
	4	Have you felt that your breath has been stale because of problems with your teeth, mouth, or dental prosthesis?	0.472/1.00	0.380/1.00	− 0.016/0.000	0.714/2.00	0.750/2.00	0.130/0.000	0.467/1.00	0.154/1.00	− 0.263/0.750
	5	Have you felt that your sense of taste has worsened because of problems with your teeth, mouth, or dental prosthesis?	0.386/1.00	0.339/1.00	− 0.033/0.000	0.605/1.50	0.575/1.00	0.000/0.000	0.100/2.00	0.077/1.75	− 0.071/0.000
	6	Have you felt that your digestion has worsened because of problems with your teeth, mouth, or dental prosthesis?	0.307/1.00	0.242/0.250	− 0.101/0.000	0.568/1.00	0.544/1.00	0.000/0.000	0.375/1.00	0.500/1.00	0.056/0.000
	12	Have you felt that there has been less flavor in your food because of problems with your teeth, mouth, or dental prosthesis?	0.312/1.00	0.254/1.00	− 0.030/0.000	0.532/1.00	0.591/1.00	− 0.019/0.000	0.286/1.00	0.273/1.75	0.000/0.000
	19	Have you had to interrupt meals because of problems with your teeth, mouth, or dental prosthesis?	0.121/0.000	0.071/0.000	9.75/0.000	0.217/1.00	0.229/1.00	9.47/2.00	0.286/1.00	0.333/1.00	9.47/1.75
	22^Φ^	Have you felt that it took longer to finish a meal?	0.160/0.250	0.135/0.000	0.017/0.000	0.400/1.00	0.409/1.00	0.020/0.000	0.846/2.75	1.17/3.00*	0.154/1.00
	25	Have you found it uncomfortable to eat any foods because of problems with your teeth, mouth, or dental prosthesis?	0.340/1.00	0.412/1.00	0.000/0.000	0.703/2.00	0.687/2.00	− 0.077/0.000	0.818/2.75	1.00/3.75	0.071/1.75
	33	Have you had to avoid eating some foods because of problems with your teeth, mouth, or dental prosthesis?	0.400/1.00	0.155/1.00	− 0.030/0.000	0.575/2.00	0.625/2.00	− 0.020/0.000	0.294/2.75	1.29/3.00*	0.000/1.50
	35	Has your diet been unsatisfactory because of problems with your teeth, mouth, or dental prosthesis?	0.210/0.000	0.210/0.000	0.061/0.000	0.535/1.50	0.595/2.00	0.127/0.000	0.357/1.00	0.429/1.75	− 0.053/0.000
	48	Have you had food catching in your teeth or dental prosthesis?	0.319/1.00	0.359/2.00	0.164/0.000	0.342/1.00	0.483/2.00	0.112/1.00	0.500/2.00	0.750/2.75*	0.294/1.00
	51	Have you had the feeling in the past 7 days that your dental prosthesis does not fit properly?	-	-	-	0.909/2.00	0.839/2.00	0.120/1.00	1.17/3.00	1.75/3.50	0.385/1.00
	53	Has it happened in the past 7 days that you were unable to eat with your dental prosthesis because of problems with it?	-	-	-	0.561/1.00	0.500/1.00	− 0.020/0.000	0.600/2.00	0.667/2.50	0.125/0.000
Psychosocial impact	2	Have you had trouble pronouncing any words because of problems with your teeth, mouth, or dental prosthesis?	0.164/0.000	0.177/0.000	0.000/0.000	0.457/1.00	0.391/1.00	0.018/0.000	0.267/1.00	0.333/1.00	0.177/0.750
	7	Have you had trouble getting along with other people because of problems with your teeth, mouth, or dental prosthesis?	0.106/0.000	0.078/0.000	− 0.015/0.000	0.192/0.500	0.265/1.00	0.055/0.000	0.053/0.000	0.056/0.000	0.000/0.000
	8	Have you suffered any financial loss because of problems with your teeth, mouth, or dental prosthesis?	0.071/0.000	0.088/0.000	− 0.016/0.000	0.231/0.000	0.217/1.00	− 0.038/0.000	− 0.167/0.000	− 0.059/0.000	0.056/0.000
	9	Have you felt that life in general was less satisfying because of problems with your teeth, mouth, or dental prosthesis?	0.271/1.00	0.246/0.250	0.016/0.000	0.568/1.00	0.634/2.00	0.018/0.000	0.461/1.75	0.615/2.00	0.059/0.000
	11	Have you found it difficult to relax because of problems with your teeth, mouth, or dental prosthesis?	0.267/1.00	0.232/0.000	0.064/0.000	0.460/1.00	0.447/1.00	− 0.019/0.000	0.368/1.00	0.389/1.00	0.000/0.000
	13	Have you felt tense because of problems with your teeth, mouth, or dental prosthesis?	0.200/1.00	0.224/1.00	− 0.032/0.000	0.440/1.00	0.553/1.00	0.098/0.000	0.167/1.00	0.667/2.00	0.333/1.00
	14	Has your speech been unclear because of problems with your teeth, mouth, or dental prosthesis?	0.220/0.250	0.172/0.000	− 0.057/0.000	0.400/1.00	0.531/1.00	0.143/0.000	0.231/1.00	0.182/1.75	0.125/0.750
	16	Have you been unable to enjoy other people’s company as much because of problems with your teeth, mouth, or dental prosthesis?	0.212/0.000	0.141/0.000	− 0.103/0.000	0.404/1.00	0.348/1.00	− 0.058/0.000	0.143/1.00	0.250/1.00	0.056/0.000
	20	Has your sleep been interrupted because of problems with your teeth, mouth, or dental prosthesis?	0.123/0.000	0.056/0.000	− 0.032/0.000	0.348/1.00	0.356/1.00	0.039/0.000	− 0.105/0.000	− 0.118/0.000	0.000/0.000
	21^Φ^	Have you had to avoid eating together with other people?	0.078/0.000	0.048/0.000	− 0.015/0.000	0.277/1.00	0.227/1.00	− 0.038/0.000	0.125/0.000	0.235/0.750	0.105/0.000
	23	Has your concentration been affected because of problems with your teeth, mouth, or dental prosthesis?	0.138/0.000	0.113/0.000	0.000/0.000	0.440/1.00	0.467/1.00	0.036/0.000	0.063/0.000	0.063/0.000	0.000/0.000
	24	Have you been unable to work to your full capacity because of problems with your teeth, mouth, or dental prosthesis?	0.246/1.00	0.179/0.000	− 0.048/0.000	0.380/1.00	0.400/1.00	0.059/0.000	0.059/0.000	0.000/0.000	− 0.111/0.000
	26	Have you felt depressed because of problems with your teeth, mouth, or dental prosthesis?	0.217/0.500	0.194/0.000	0.000/0.000	0.447/1.00	0.534/1.50	0.056/0.000	0.000/0.000	0.000/0.000	0.000/0.000
	27	Have you been a bit irritable with other people because of problems with your teeth, mouth, or dental prosthesis?	0.175/0.000	0.131/0.000	− 0.045/0.000	0.447/1.00	0.419/1.00	− 0.039/0.000	0.063/0.000	0.056/0.000	0.000/0.000
	28	Have you avoided going out because of problems with your teeth, mouth, or dental prosthesis?	0.125/0.000	0.065/0.000	− 0.074/0.000	0.240/0.000	0.260/1.00	0.017/0.000	0.167/0.000	− 0.059/0.000	− 0.105/0.000
	29	Have you been less tolerant of your partner or family because of problems with your teeth, mouth, or dental prosthesis?	0.184/0.000	0.158/0.000	0.015/0.000	0.296/1.00	0.378/1.00	0.172/0.000	0.125/0.000	0.186/0.750	0.053/0.000
	30	Have you had difficulty doing your usual jobs because of problems with your teeth, mouth, or dental prosthesis?	0.188/0.000	0.164/0.000	− 0.030/0.000	0.306/1.00	0.298/1.00	− 0.018/0.000	0.133/0.000	0.186/0.000	0.056/0.000
	31	Have you been totally unable to function because of problems with your teeth, mouth, or dental prosthesis?	0.186/0.000	0.164/0.000	− 0.015/0.000	0.204/0.500	0.143/0.000	− 0.037/0.000	0.353/1.00	0.267/1.00	− 0.211/0.000
	32	Have you been a bit embarrassed because of problems with your teeth, mouth, or dental prosthesis?	0.167/0.000	0.155/0.000	− 0.016/0.000	0.432/1.00	0.391/1.00	− 0.056/0.000	0.294/0.750	0.000/1.50	− 0.263/0.750
	34	Have you been upset because of problems with your teeth, mouth, or dental prosthesis?	0.164/0.000	0.195/0.000	− 0.088/0.000	0.438/1.00	0.381/1.00	− 0.125/0.000	0.000/0.000	0.118/0.000	0.000/0.000
	43	Have you been self-conscious because of your teeth, mouth, or dental prosthesis?	0.312/1.00	0.263/1.00	0.000/0.000	0.500/2.00	0.514/2.00	0.135/0.000	0.375/1.00	0.438/1.00	0.059/0.000
	44	Have dental problems made you miserable?	0.206/0.000	0.207/0.000	0.048/0.000	0.392/1.00	0.313/1.00	0.000/0.000	0.000/0.000	0.000/0.000	− 0.053/0.000
	50	Have you been worried about dental problems?	0.364/1.00	0.426/1.00	0.039/0.000	0.579/2.00	0.639/2.00	0.128/0.000	0.429/1.00	0.500/1.00	0.067/0.000
Linguistic limitations	15	Have people misunderstood some of your words because of problems with your teeth, mouth, or dental prosthesis?	0.141/0.000	0.131/0.000	0.015/0.000	0.360/1.00	0.438/1.00	0.098/0.000	0.313/1.00	0.250/1.00	− 0.063/0.000
	17	Have you been unable to brush your teeth properly because of problems with your teeth, mouth, or dental prosthesis?	0.309/0.000	0.396/1.00	0.082/0.000	0.617/1.00	0.767/2.00	0.135/0.000	0.444/2.00	0.400/2.00	− 0.059/0.000
	36	Have you had headaches because of problems with your teeth, mouth, or dental prosthesis?	0.100/0.000	0.125/0.000	0.032/0.000	0.196/0.000	0.279/1.00	0.061/0.000	0.105/0.000	0.059/0.000	− 0.059/0.000
Orofacial pain	37	Have you had painful aching in your mouth?	0.278/1.00	0.429/1.00	0.172/0.000	0.182/1.00	0.477/1.00	0.413/1.00	0.182/1.75	0.417/1.75	0.182/1.75
	38	Have you had a sore jaw?	0.264/1.00	0.407/1.00	0.136/0.000	− 0.119/1.50	0.319/1.00	0.400/1.00	0.300/2.00*	0.273/1.75	0.125/0.750
	39	Have you had sensitive teeth, for example, due to hot or cold foods or drinks?	0.167/1.00	0.268/1.00	0.000/0.000	0.273/1.00	0.525/1.50	0.228/1.00	0.267/1.00	0.231/1.00	0.000/2.00
	40	Have you had a toothache?	0.146/0.250	0.109/0.000	0.018/0.000	0.174/0.750	0.170/0.000	0.020/0.000	0.158/0.000	0.000/2.00	− 0.158/0.000
	41	Have you had painful gums?	0.400/1.00	0.456/1.00	0.000/0.000	0.237/1.00	0.419/1.00	0.250/1.00	0.077/0.750	0.214/1.00	0.143/0.750*
	42	Have you had sore spots in your mouth?	0.309/1.00	0.367/1.00	0.081/0.000	− 0.024/2.00	0.267/1.00	0.386/1.00	0.400/2.00*	0.539/1.00	0.417/1.00
	45^Φ^	Have you had a disturbing noise in your jaw?	0.065/0.000	0.462/0.000	− 0.047/0.000	0.154/0.000	0.094/0.000	− 0.039/0.000	0.059/0.000	0.059/0.000	− 0.111/0.000
	46^Φ^	Have you had an unpleasant dry mouth?	0.233/1.00	0.224/0.000	0.032/0.000	0.167/2.00	0.477/1.00	0.340/1.00	0.923/1.75	0.692/1.75	− 0.063/0.000
	52	Has your dental prosthesis pressed uncomfortably in the past 7 days?	-	-	-	0.333/1.00	0.474/1.00	0.216/1.00	− 0.300/1.75	0.636/1.75	0.643/1.00

^Φ^Only part of the German version of the Oral Health Impact Profile (OHIP-G53)

^*^Data normally distributed

### Statistical analysis

The individual difference values for the three investigation periods (T0–T1; T0–T2; T1–T2) and the corresponding dimensions were evaluated separately. The individual replies at times T0, T1, and T2 were considered. If the patient gave the same answer of value 0–5, the question was defined with the value 0; if the patient gave a positive or negative answer in the following questionnaire, a − or + was defined with the corresponding number, such as + 1 or − 3 [15].

The normal distribution was tested by Kolmogorov–Smirnov test. Significant differences in the questionnaires with respect to investigation times and dimensions were analyzed with Wilcoxon’s signed rank for matched pairs, Kruskal–Wallis, and Mann–Whitney *U* tests using the grouped medians. Cronbach’s alpha test was performed as an additional reliability analysis to secure the compilation of all questions to the various dimensions. A power analysis was performed for all subgroups with the software G*Power 3.1 (HHU Düsseldorf, Germany). Data were analyzed with the SPSS version 25.0 (SPSS, Chicago, IL, USA), and the level of significance was set at *p* = 0.017.

## Results

The mean age of the patients was 64.7 ± 10.51 years.

Among the questions, 94.9% and among the dimensions 82.2% of the values did not show a normal distribution. Consequently, non-parametric tests were subsequently used.

The Cronbach’s alpha test revealed values between 0.738 (linguistic limitations) and 0.966 (psychological impact) within the five dimensions.

For question 3/10 (appearance) and question 1 (oral function), the greatest improvement was observed between T0 and T1 (median 0.971) and for question 1 also between T0 and T2 (median 1.304), followed by question 46 (orofacial pain) between T0 and T1 (median 0.923) and question 51 (oral function) between T0 and T1 (median 0.909).

The power analysis showed a power of over 90% for the individual subgroups of the different restoration types and dimensions for fixed restorations and removable dental prostheses, with the exception of the dimension of oral pain for removable dental prostheses (39%). For the restoration type of telescopic dental prostheses, the power ranged between 41 and 77%.

### Differences between investigation times within the five dimensions

**Fig. 2:**
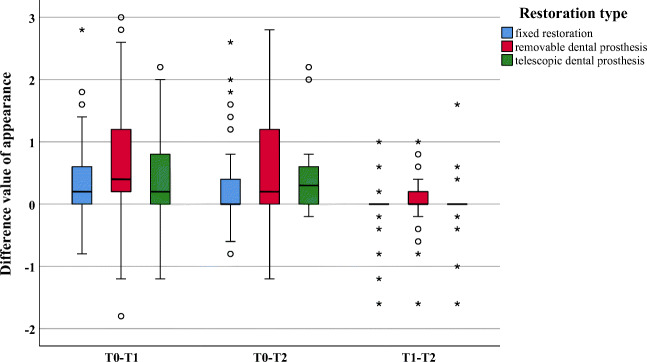
Boxplot of difference values for the dimension appearance

**Fig. 3:**
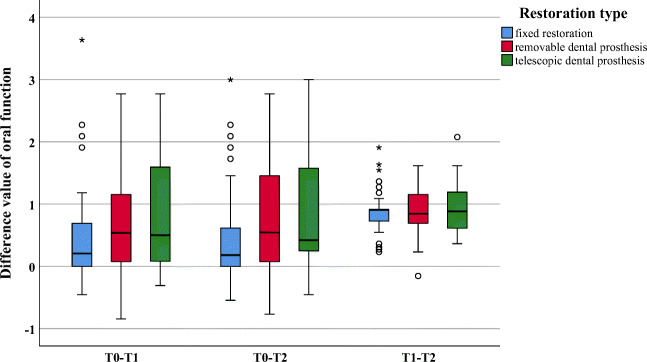
Boxplot of difference values for the dimension oral function

Within the dimensions “appearance” and “oral function,” significant improvements were observed between investigation times T0 and T1 (appearance *p* = 0.012; oral function *p* = 0.029) and between T0 and T2 (*p* = 0.005 and *p* = 0.013, respectively). No significant difference could be found between investigation times T1 and T2 (appearance *p* = 0.261; oral function *p* = 0.983) (Figs. 2 and 3).

**Fig. 4:**
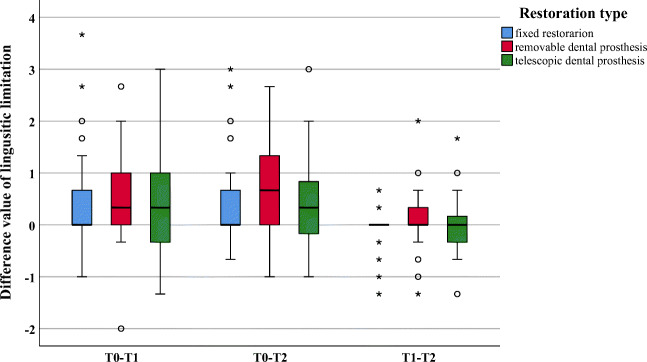
Boxplot of difference values for the dimension linguistic limitation

Within the dimension “linguistic limitations,” significant improvement was only observed between T0 and T2 (*p* = 0.008); no significant differences were found between the other investigation times (*p* ≥ 0.067) (Fig.4).

**Fig. 5:**
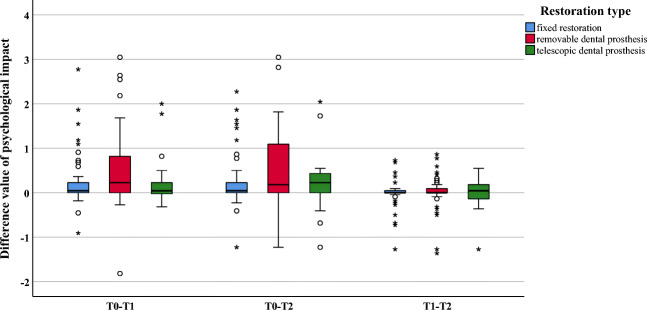
Boxplot of difference values for the dimension orofacial pain

Within the dimension “orofacial pain,” a significant improvement was found between T1 and T2 (*p* = 0.012). The other investigation times did not show any differences (*p* ≥ 0.537) (Fig. 5).

**Fig. 6:**
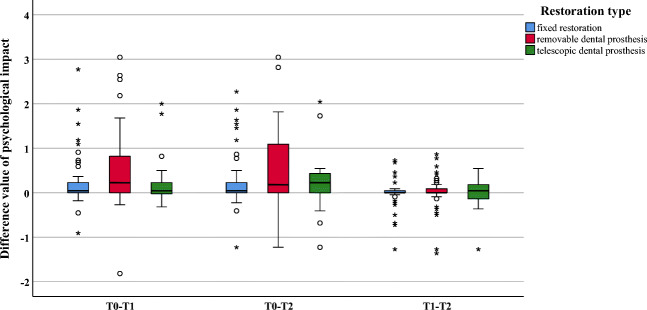
Boxplot of difference values for the dimension psychological impact

No significant differences were found within the dimension “psychological impact” (*p* ≥ 0.051) (Fig. 6).

### Differences between the dental prostheses within the investigation times

Within the investigation period T0–T1, significantly greater improvements were found for removable as compared with fixed restorations for the dimensions “oral function” (*p* = 0.012), “linguistic limitations” (*p* = 0.016), and “appearance” (*p* = 0.003) (Figs. 2, 3, and 4). All other dimensions (*p* ≥ 0.023) presented no changes (Figs. 5 and 6). In addition, no significant difference was found between fixed and telescopic prostheses (*p* ≥ 0.104) or between removable and telescopic dental prostheses (*p* ≥ 0.100).

Within the investigation period T0–T2, significantly greater improvements were found for removable dental prostheses compared with fixed ones for “oral function” (*p* = 0.014), “linguistic limitations” (*p* = 0.002), and “appearance” (*p* = 0.001) (Figs. 2, 3, and 4). All other dimensions (*p* ≥ 0.024) presented no changes (Figs. 5 and 6). In addition, no significant difference was found between fixed and telescopic dental prostheses (*p* ≥ 0.020) or between removable and telescopic dental prostheses (*p* ≥ 0.158).

Within the investigation period T1–T2, a significant improvement (*p* = 0.007) could only be found for the dimension “orofacial pain”; all others showed no significant differences (*p* ≥ 0.100) (Fig. 5).

## Discussion

The first hypothesis of the present study, which stated that there is no change in patients’ oral health-related impact profile after prosthetic treatment in a clinical student course in relation to specific questions and dimensions, can be rejected. Patients showed partly significant improvements in oral health-related impact profile after prosthetic treatment in a student course. In addition, significant differences were shown between the types of dental prosthesis in terms of oral health-related impact profile. Consequently, the second hypothesis can also be rejected. The specific differences are discussed below.

The OHIP-G49 for fixed and OHIP-G53 for removable dental prostheses were used in the present study to analyze five different dimensions with corresponding questions. In particular, the important dimension “appearance” is not considered in the shorter OHIP versions [8, 15]. Since all parameters were essential for the present study and the dimension “appearance” did not play a superordinate role, no additional esthetic modulus was used, as is sometimes recommended in the literature [8].

Regarding fixed restorations, the present results showed a significantly lower increase in the OHRQoL in patients with fixed restorations than in those with removable dental prostheses for the dimensions “oral function,” “linguistic limitations,” and “appearance” at both T1 and T2 in relation to the baseline values, but the initial situation is decisive in this context. Most of the patients who received fixed restorations had no previous prosthetic restoration. The improvement of the value therefore seems to be lower. In contrast, the improvement of the OHRQoL with new removable dental prostheses in patients who had no prosthetic restoration before or new removable dental prostheses that probably have a better fit seem obvious. As in the present study, the prosthetic treatment with fixed restorations showed an improvement in OHRQoL in the scientific literature and data from other studies analyzing single-tooth restorations or multiple-unit fixed dental prostheses [19–21].

Nevertheless, all restoration types showed an improvement as already described in literature [22]. Within the dimension “appearance,” a significant improvement was identified between the baseline values T0 and T1. The greatest improvement was found for the question of whether the patients felt esthetically impaired. After insertion of the prosthetic restoration, there was no significant change within the values (T1 to T2). The patients thus seemed to evaluate the esthetic parameters as the same or similar to those after 3 months of intraoral use. It seems that the patient’s assessment and perception of the esthetic change can be determined directly at the follow-up appointment.

The same results were measured for the dimension “oral function.” In particular, question 51, which is specifically for removable dental prostheses, including telescopic restorations, and asks whether patients feel that their dental prosthesis has not fitted properly in the last 7 days, showed the greatest improvements. This can be explained by the fact that this is probably one of the main reasons for prosthetic treatment with new removable dental prostheses.

A significant improvement in “linguistic limitations,” on the other hand, could only be found between T0 and T2 and was not already apparent at T1. Patients, especially those newly treated with removable dental prostheses with possibly palatal coverage, need time to get used to the linguistic function, which is underlined by the present results [23, 24]. Consequently, at investigation time T2, clear improvements in contrast to the pre-prosthetic situation were evident, but the underlying pre-prosthetic situation indeed played a major role [25].

In addition, a delayed improvement in the dimension of “orofacial pain” was only observed at investigation time T2 in relation to the data obtained at the follow-up appointment directly after insertion, regardless of the restoration type. This could also be due to the time required for any tissue or muscular structures to facilitate a specific impairment. Within this dimension, the greatest improvements for patients seem to have been achieved in the area of dry mouth.

In the study of Jenei et al., the type of dental prosthesis showed no significant impact on the overall OHIP score [7]. However, in that study, the overall OHIP score was analyzed, but the individual questions were not divided into individual dimensions. Consequently, a direct comparison is impossible.

Viola et al. found that the scores improved in patients treated with complete dental prostheses [17]. In general, there seemed to be no difference in OHRQoL between partial and complete dentures [26]. The diverse occlusal concepts were further comparable in the literature [27]. Patient satisfaction with prosthetic care had a positive influence on OHRQoL and the daily life of patients [28]. It could be shown that oral health-related impact profile in patients treated with a removable dental prosthesis is significantly influenced by educational level, socioeconomic status, health status, and cigarette consumption [29]. These parameters, however, were not queried and correlated in the present study.

Regarding telescopic dental prostheses, there seemed to be no difference in oral health-related impact profile when patients were treated with telescopic dental prostheses compared with removable dental prostheses or fixed restorations. The literature also showed an improvement of the oral health-related impact profile, also for different telescope types [18, 30]. However, it must be noted that the power analysis with only 20 telescopic prostheses was in a low range between 41 and 77% compared with the 61/70 patients of the other two groups with a power more than 90%. These results must therefore be interpreted with caution and a clear conclusion cannot be drawn. In order to make reliable statements, a larger group size is required.

A limitation of the present study is the long time period between T1 and T2, which was about 3 months. A measurement of the results after 1 month could have highlighted the faster improvement of the dimensions “orofacial pain” and “linguistic limitations”. Consequently, it cannot clearly be concluded how long a patient needs for an improvement. In other studies, different follow-up times were selected, but these partly correspond to the present study. The examination times are usually before the actual prosthetic treatment as a baseline value, directly after the insertion at the check-up date, and after 1 month [7, 11, 12, 15], 3 months, or 6 to 12 months [7, 12, 18]. In contrast to these longer observation periods, monitoring after 7 days also seems to be equally reliable and valid as after 1 month [31]. A limitation is certainly also the own division into five dimensions as already applied in an earlier publication [15]. In the literature, there are different methods of analysis and subdivisions of the OHIP questionnaires and also different numbers of questions within different OHIP questionnaires. This is difficult for direct comparisons in the literature. A possible standardization/calibration of the analyses could help to better compare the studies. Another limitation that may have had an influence on the results is the treatment of patients in a student course: patients have to plan considerably more time as the treatment is prolonged. The final result or the quality of the prosthetic work itself plays a rather subordinate role [32], since all steps are supervised and, if necessary, corrected by experienced dentists. On the other hand, answering the OHIP-49/53 questions represents a further limitation, as it is highly subjective and is also influenced by patients’ expectations and general satisfaction. However, the questionnaire is a tool that can measure patients’ oral health-related impact profile, even if a certain bias cannot be avoided [25]. Further investigation into the analysis of individual prosthetic restorations with the additional subdivision of removable into removal partial dental prostheses with clasps and complete dental prostheses or, in the case of fixed dental prostheses, into crowns and fixed dental prostheses, is conceivable. For this, however, a larger patient cohort would be necessary in order to be able to make the most valid statements possible.

## Conclusion

Within the limitations of the present study, the following conclusions can be drawn:

1. Significant improvements in oral health-related impact profile after fitting of dental prostheses were found between investigation times T0 and T1 and T0 and T2 for the dimensions “appearance” and “oral function,” independent of the restoration type.
2. The dimension “linguistic limitations” showed a delayed improvement in oral health-related impact profile between T0 and T2, and the dimension “orofacial pain” showed the same between T1 and T2, independent of dental prosthesis type.
3. For removable dental prostheses, a significantly greater improvement in oral health-related impact profile was found for the dimensions “oral function,” “linguistic limitations,” and “appearance” in contrast to fixed dental prostheses between T0 and T1, as well as between T0 and T2 and for “orofacial pain” from T1 to T2. However, the individual initial situations must also be taken into account.
